# Exercise intervention to prevent falls and enhance mobility in community dwellers after stroke: a protocol for a randomised controlled trial

**DOI:** 10.1186/1471-2377-9-38

**Published:** 2009-07-22

**Authors:** Catherine M Dean, Chris Rissel, Michelle Sharkey, Catherine Sherrington, Robert G Cumming, Ruth N Barker, Stephen R Lord, Sandra D O'Rourke, Catherine Kirkham

**Affiliations:** 1 Discipline of Physiotherapy, Faculty of Health Sciences, The University of Sydney, PO Box 170, Lidcombe, NSW 1825, Australia; 2 Health Promotion Service, Sydney South West Area Health Service, Level 9 (North), King George V Missenden Road, Camperdown, NSW 2050, Australia; 3 School of Public Health, The University of Sydney, Sydney, Australia; 4 Stroke Recovery Association NSW, PO Box 3401, Putney, NSW 2112, Australia; 5 Musculoskeletal Division, The George Institute for International Health, The University of Sydney, PO Box M201, Missenden Rd, Sydney, NSW 2050, Australia; 6 Centre for Education and Research on Ageing, Concord Hospital, Sydney, Australia; 7 School of Public Health, Tropical Medicine and Rehabilitation Sciences, James Cook University, Townsville, QLD 4811, Australia; 8 Prince of Wales Medical Research Institute, The University of New South Wales, PO Box 82, St Pauls, NSW 2031, Australia

## Abstract

**Background:**

Stroke is the most common disabling neurological condition in adults. Falls and poor mobility are major contributors to stroke-related disability. Falls are more frequent and more likely to result in injury among stroke survivors than among the general older population. Currently there is good evidence that exercise can enhance mobility after stroke, yet ongoing exercise programs for general community-based stroke survivors are not routinely available. This randomised controlled trial will investigate whether exercise can reduce fall rates and increase mobility and physical activity levels in stroke survivors.

**Methods and design:**

Three hundred and fifty community dwelling stroke survivors will be recruited. Participants will have no medical contradictions to exercise and be cognitively and physically able to complete the assessments and exercise program. After the completion of the pre-test assessment, participants will be randomly allocated to one of two intervention groups. Both intervention groups will participate in weekly group-based exercises and a home program for twelve months. In the lower limb intervention group, individualised programs of weight-bearing balance and strengthening exercises will be prescribed. The upper limb/cognition group will receive exercises aimed at management and improvement of function of the affected upper limb and cognition carried out in the seated position. The primary outcome measures will be falls (measured with 12 month calendars) and mobility. Secondary outcome measures will be risk of falling, physical activity levels, community participation, quality of life, health service utilisation, upper limb function and cognition.

**Discussion:**

This study aims to establish and evaluate community-based sustainable exercise programs for stroke survivors. We will determine the effects of the exercise programs in preventing falls and enhancing mobility among people following stroke. This program, if found to be effective, has the potential to be implemented within existing community services.

**Trial registration:**

The protocol for this study is registered with the Australian New Zealand Clinical Trials Registry (ACTRN12606000479505).

## Background

Stroke is a leading cause of death and disability. The annual estimated direct and indirect costs of stroke in the US in 2009 is $68.9 billion [1]. The major burden of stroke is chronic disability rather than death [2]. It is projected that stroke-related disability will increase dramatically over the next two decades as the population ages [3]. Development and implementation of effective strategies to minimise stroke-related disability is essential to minimise future health costs.

On completion of rehabilitation most stroke survivors can walk independently, however 90% of these people are unable to walk with sufficient speed or endurance to function effectively in the community [4] and 70% of stroke survivors will fall within six months of discharge from hospital [5]. Gait and balance problems have been found to be important risk factors for falls in stroke survivors [6,7]. Walking speed and walking capacity among community dwelling stroke survivors is markedly lower than age-matched controls [8,9], which results in major limitations of community participation.

Stroke typically affects older persons. The development of effective strategies to minimise disability and falls among older people is an urgent public health challenge due to the increase of older people in the global population. Around 500 million people worldwide are aged 65 and older and this number is predicted to increase to 1 billion by 2020 [10]. Falls adversely affect the quality of life of older people and can have serious consequences including fractures. Stroke survivors are four times more likely to suffer a hip fracture than other community-dwellers [11].

Systematic reviews in the general older population have found that exercise programs which specifically target balance and lower limb muscle strength are effective in preventing falls [12,13]. It is less clear whether exercise can prevent falls among people following stroke. There is also good evidence that well-designed exercise programs can enhance functional abilities after stroke [14]. Previous work has shown the effectiveness of small-group "circuit-style" exercise programs among people following stroke [15] or other disabling conditions [16] for improving mobility. Previously it was thought that recovery following stroke occurs within the first three months and is complete by 12 months [17]. However there is growing evidence that functional abilities can improve with exercise many years after stroke [9,18,19]

The Weight-bearing Exercise for Better Balance (WEBB) exercise program has been developed by two of the authors (CS and CMD) and colleagues to specifically target poor balance and lower limb muscles weakness for people at risk of falls. The main aim of this randomised controlled trial is to determine the effectiveness of the WEBB exercise program on reducing falls and enhancing mobility among community dwelling stroke survivors. In addition we will measure the effects of the program on risk of falling, physical activity levels, community participation, quality of life and health service utilisation. The WEBB program is available on request from the first author. A secondary aim of the trial is to evaluate the effect of the upper limb/cognitive intervention program, on upper limb function and cognition.

## Methods/design

### Design

**Figure 1:**
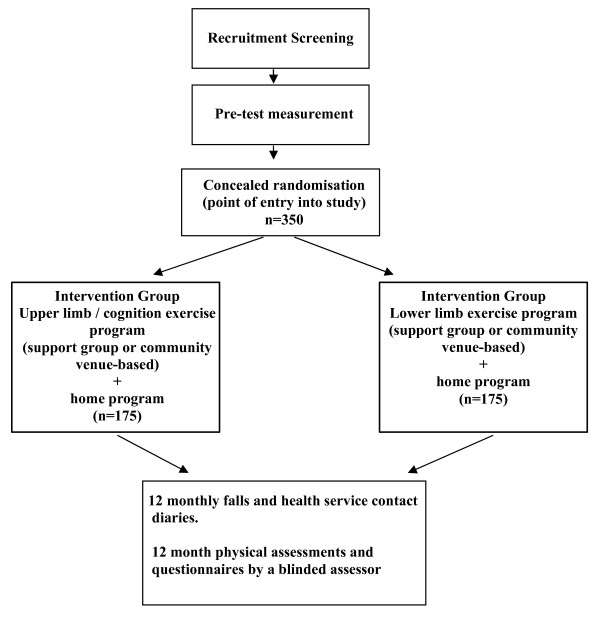
**Trial design**.

A randomised controlled trial will be conducted with three hundred and fifty community-dwelling stroke survivors (Figure 1).

### Participant selection

Participants will be randomly assigned to one of two intervention groups and will be primarily recruited through the Stroke Recovery Association of New South Wales (NSW), Australia support group (Stroke Club) network. This Association holds regular Stroke Club meetings throughout NSW. The exercise classes will be conducted weekly in the venues used by the Stroke Club, prior to and after meeting times. It is anticipated that each Stroke Club will have participants randomised into both intervention groups and both types of classes will be offered at different times at the same venue. Additional participants will be recruited through advertisement and local physiotherapists and will attend the same exercise classes. To be eligible for the study, participants must be community-dwelling stroke survivors aged 40 years and above and be able to walk 10 m independently with or without a mobility aid.

Participants will be excluded if they have a Mini-Mental State Examination score [20] of < 20, have insufficient communication skills to participate in assessment and intervention, have a medical condition which precludes exercise (eg. unstable cardiovascular disease) or suffer from other uncontrolled chronic conditions that would interfere with the safety and conduct of the training and testing protocol.

All potential volunteers will be screened for eligibility by a physiotherapist. Medical clearance will be required from each participant's medical practitioner to certify him/her able to participate in moderate-intensity semi-supervised exercise before being accepted into the trial.

The study protocol has been approved by Sydney South West Area Health Service Ethics Committee (Clearance No: X06-0039) and The University of Sydney Human Research Ethics Committee (HREC Number 07/2006/9031), and written informed consent will be obtained from all participants.

### Measurements and Procedures

All participants will undergo two measurements: one on entry to the study (pre-test) and one after the 12-month period (post-test) by a physiotherapist blinded to group allocation. All pre-test measures will be completed prior to randomisation.

### Randomisation

After approval from the participant's usual General Practitioner and completion of the initial assessment, participants will be formally entered into the study and randomised to either the lower limb intervention or upper limb/cognition intervention group. Randomisation will be stratified by stroke club site using a computer generated random number schedule with variable block sizes 2–6. Generation of the randomisation sequence will be performed centrally by a researcher not involved in recruitment or assessment. Group allocation will be concealed using consecutively-numbered opaque envelopes. The opaque envelope will be opened after completion of the assessment in the presence of the participant.

## Intervention groups

Both intervention groups will receive weekly exercise classes and a home program for twelve months. The home programs will be reviewed monthly by a physiotherapist and modified if necessary.

### Lower limb group

The lower limb intervention group will receive an exercise intervention designed to prevent falls, enhance mobility and increase physical activity. This will involve a weekly circuit-style group exercise class, a home exercise program and a walking program. Interventions will be tailored to an individual's functional ability and the nature and difficulty of exercises will be progressed regularly.

The lower limb group will undertake exercises from the WEBB program. Participants will participate in a weekly exercise classes conducted by a physiotherapist and will be asked to perform a home exercise program at least 3 times a week. The home program and exercise classes include a 45–60 min program of progressive balance, lower limb strengthening exercises and walking. The exercise classes and home program will include 5 minutes warm-up exercises. The lower limb extensor muscle groups, which act to prevent collapse of the lower limb (hip and knee extensors and ankle plantarflexors) will be targeted with exercises designed to enhance balance and muscle strength. The balance exercises include standing with a decreased base of support, forwards and sideways stepping/walking, and graded reaching activities in standing. Strengthening exercises will include sit-to-stand, forward or lateral step-ups onto a small block, semi squats and heel raises in standing. Resistance for strengthening exercises will be applied using weighted vests in a similar protocol to one which has been successfully used by people with multiple sclerosis in a home-based program [21]. Standard principles governing frequency, volume, duration, intensity and progression of exercise will be applied [22].

### Upper limb/cognition group

The upper limb/cognition intervention group will receive a weekly exercise class aimed at management and improved function of the affected upper limb and cognition, carried out in the seated position. They will also be given a home exercise program. The upper limb component of the intervention was designed and developed by one of authors RB. We postulate that this intervention may lead to improvements in upper limb function and cognition but is unlikely to affect change in falls and mobility. This study design will, therefore, enable assessment of additional benefits of the lower limb intervention over and above likely benefits of social aspects of exercise classes.

### Safety

Participants will be instructed how to perform exercises safely with a stable object (such as a table) located nearby for additional support if required. Participants will be provided with a booklet containing safety precautions, instructions and photographs of exercises for use in exercise sessions at home. In addition, they will be provided with a logbook for recording exercises completed, effects of exercise (e.g. muscle soreness). Where appropriate, family members and/or carers, will be encouraged to assist with supervision and performance of the exercise program.

### Outcome measures

The primary outcome measures will be falls and mobility. Falls will be defined as "events that resulted in a person coming to rest unintentionally on the ground or other lower level, not as the result of a major intrinsic event or an overwhelming hazard" [23]. Falls will be assessed by comparing the number of falls in intervention and control groups. The proportion of fallers in each group will also be compared. Falls will be monitored for one year with monthly fall calendars. All participants will receive monthly calendars on entry to the study, with instructions to record the following events: number of falls, visits to or by nursing and allied health personnel, general practitioner or specialists appointments and hospitalisations. Participants will be asked to return the completed calendar monthly at their weekly exercise class. If calendars are not returned, further contact will be made by telephone. Details of any falls (including how and where the fall occurred, injuries suffered, medical intervention required and limitations to activity as a result of a fall) will be verified.

Two aspects of mobility, walking speed and capacity will be assessed. Walking speed will be measured using the 10 m walk test. Participants will be timed as they walk at their comfortable speed over the middle 10 m of a level 14 m walk track. Walking capacity will be measured by the distance covered in the 6 min walk test. The test will be conducted over a 25 – 30 m level corridor and standardised instructions will be given to each participant prior to the test and standardised encouragement at each minute in accordance with the American Thoracic Society guidelines for the 6 min walk test [24]. For safety, while administering this test, the assessor will walk slightly behind, and not beside, the participant so as to avoid influencing the participant's self selected walking pace.

Secondary outcome measures listed below will be collected on entry and at the end of the 12-month study period. These outcome measures will be

i) Falls risk assessed using the Physiological Profile Assessment [25]

ii) Physical activity assessed via a 7-day pedometer count

iii) Community participation using the Adelaide activity profile [26]

iv) Quality of life and well-being using the SF 12™ Version 2

v) Health service utilisation using monthly calendars

vi) Upper limb function using Items 6,7,8 of Motor Assessment Scale for Stroke [27] and the nine hole peg test [28]

vii) Cognition using the Montreal Cognitive Assessment [29].

### Statistical analysis

The number of falls per person-year will be analysed using negative binomial regression to estimate the difference in fall rates between the two groups, adjusted for previous multiple faller status [30]. The proportion of fallers between groups will be compared using the relative risk statistic. Between-group comparisons of final test performance for the continuously-scored outcome measures will be made using General Linear Models (ANCOVA) controlled for pre-test performance. Ordinally-scored data will be analysed for between group differences using the non-parametric Mann Whitney U statistic. An intention-to-treat approach will be used for all analyses.

### Sample size

As fall rates will be compared between groups using incident rate ratios (IRR) from negative bionomial regression models [30] we conducted an analysis of statistical power using the nbpower command in the STATA software package. A total of 350 participants (175 per group) will be recruited. The study will have 80% power to detect as significant at the 5% level a 34% reduction in the rate of falling (i.e. an IRR of 0.66 using negative binomial regression analysis) in the 12 month follow-up period. This allows for a 15% loss to follow-up due to death or withdrawal from the study. Experience from our previous work indicates that, with this sample size, power will also be sufficient to detect meaningful between-group differences for all other outcome measures [9,15,16].

## Discussion

It is widely accepted that in order to maintain health, physical activity is essential. Currently in the study area, routine provision of long term ongoing physical activity/exercise programs for people living in the community after stroke is virtually non-existent. There are a range of community-based physical activity programs for healthy older people. However, the chronic disability often associated with stroke means that these programs are not usually accessible for this population.

There is an urgent need to identify cost-effective evidence-based interventions for reducing falls and related injuries in people who has had a stroke. The WEBB program has been designed for falls prevention and so includes high challenge, yet safe, progressive balance exercises targeting standing balance and walking as well as progressive moderate to high intensity leg strengthening exercises with resistance applied using weighted vests. The program is currently also being evaluated in older persons recently discharged from hospital [31] and people with Parkinson's disease [32].

This project aims to investigate the effects of the WEBB program in community dwelling stroke survivors using a randomised controlled trial incorporating the features known to reduce bias (i.e. concealed random allocation to groups, blinded outcome assessment and intention to treat analysis) and large enough to detect any effect of the intervention on fall rates. This project will determine the effectiveness of the exercise intervention in reducing falls and enhance physical activity. In addition, we will also provide new knowledge about the potential for collaborating with existing community support groups in the provision of physical activity programs. Due to the partnership with NSW Stroke Recovery Association and their extensive network of stroke clubs, there is good potential for the proposed program to be incorporated into usual Stroke Club activities at the completion of the study period, if it is found to be effective. If this community-based exercise intervention is effective, this approach of utilising support groups could also have wide applications for other chronic diseases which are worsened by inactivity.

## Competing interests

The authors declare that they have no competing interests.

## Authors' contributions

This manuscript was drafted by CD, CK, CS and SO. The other authors have also been actively involved in the development of this study protocol. Many contributed to the writing of the grant application for this project which formed the basis for this manuscript. All authors contributing to its critical review and approving the final draft.

## Pre-publication history

The pre-publication history for this paper can be accessed here:


